# Phase Transformations of α-Alumina Made from Waste Aluminum via a Precipitation Technique

**DOI:** 10.3390/ijms131216812

**Published:** 2012-12-07

**Authors:** Khamirul Amin Matori, Loy Chee Wah, Mansor Hashim, Ismayadi Ismail, Mohd Hafiz Mohd Zaid

**Affiliations:** 1 Department of Physics, Faculty of Science, Universiti Putra Malaysia, UPM Serdang, Selangor 43400, Malaysia; E-Mails: master_loy_41f@hotmail.com (L.C.W.); mansor@science.upm.edu.my (M.H.); mhmzaid@gmail.com (M.H.M.Z.); 2 Materials Synthesis and Characterization Laboratory, Institute of Advanced Technology, Universiti Putra Malaysia, UPM Serdang, Selangor 43400, Malaysia; E-Mail: ismayadi@putra.upm.edu.my

**Keywords:** precipitation, calcinations, phase transformations, α-alumina

## Abstract

We report on a recycling project in which α-Al_2_O_3_ was produced from aluminum cans because no such work has been reported in literature. Heated aluminum cans were mixed with 8.0 M of H_2_SO_4_ solution to form an Al_2_(SO_4_)_3_ solution. The Al_2_(SO_4_)_3_ salt was contained in a white semi-liquid solution with excess H_2_SO_4_; some unreacted aluminum pieces were also present. The solution was filtered and mixed with ethanol in a ratio of 2:3, to form a white solid of Al_2_(SO_4_)_3_·18H_2_O. The Al_2_(SO_4_)_3_·18H_2_O was calcined in an electrical furnace for 3 h at temperatures of 400–1400 °C. The heating and cooling rates were 10 °C/min. XRD was used to investigate the phase changes at different temperatures and XRF was used to determine the elemental composition in the alumina produced. A series of different alumina compositions, made by repeated dehydration and desulfonation of the Al_2_(SO_4_)_3_·18H_2_O, is reported. All transitional alumina phases produced at low temperatures were converted to α-Al_2_O_3_ at high temperatures. The X-ray diffraction results indicated that the α-Al_2_O_3_ phase was realized when the calcination temperature was at 1200 °C or higher.

## 1. Introduction

Alumina is one of the most important ceramic oxides and has a wide range of uses, including high-temperature applications and microelectronics. Alumina nano-powders are utilized in many different industries such as electronics, metallurgy, optoelectronics and fine ceramic composites [1]. Alumina, Al_2_O_3_, has a molar mass of 101.96 g mol^−1^ and a density of 3.95–4.1 g cm^−3^. Alumina has high melting and boiling points of 2072 °C and 2977 °C, respectively. Alumina also has a high compression strength, high abrasion resistance, high chemical resistance, high thermal shock resistance, high degree of refractoriness, and high dielectric strength, is transparent to microwave radio frequencies and has a low neutron cross section capture area [2].

In recent years, attention has been focused on the preparation of high-purity α-Al_2_O_3_ nano-powders by various routes such as gas phase deposition, hydrothermal synthesis [2], plasma synthesis [3], the sol-gel method [4], freeze drying of sulfate solutions [5], controlled hydrolysis of metal alkoxide [6], decomposition of organo-metallic compounds in supercritical fluids and aerosol methods [7]. Many of these techniques produce nanometer-sized particles that are either amorphous or in γ-phase. In some techniques, the alumina loses its nano-crystalline nature upon repetitive calcinations because as γ-alumina is transformed to α-alumina, it undergoes rapid grain growth [8]. This grain growth process destroys the beneficial properties of the starting nano-crystalline alumina powders. In addition to the above techniques, concentrated acids, such as sulfuric acid, hydrochloric acid and nitric acid can be reacted with aluminum to form an aluminum salt. Gonczy and Mitsche used hydrochloric and sulfuric acid in the range of 3 M to 12 M to prepare high-purity α-alumina [9]. A lower concentration acid takes a longer time and does not react completely with aluminum because the amount of heat released is smaller and the rate of reaction is lower. Highly concentrated nitric acid reacts with aluminum to form nanohydrate aluminum nitrate Al(NO_3_)_3_·9H_2_O. When the nanohydrate aluminum nitrate salt is calcined at high temperature, it decomposes to form aluminum oxide and release nitrogen dioxide gas, which is a pungent-smelling gas of reddish-brown color [10]. Highly concentrated sulfuric acid also reacts with aluminum to form octadecahydrate aluminum sulfate Al_2_(SO_4_)_3_·18H_2_O. When the salt calcined at high temperature as 360 °C, it starts to decompose, and phase transformation of the alumina starts to occur. Sulfur trioxide gas, a colorless and pungent-smelling gas, is eliminated at this stage. At 950 °C, all of the aluminum sulfate is completely decomposed to alumina [11]. Octadecahydrate aluminum sulfate has a molar mass of 666.42 g mol^−1^, a density of 1.62 g cm^−3^ and a melting point of 86.5 °C. It decomposes to anhydrous aluminum sulfate at 770 °C. It is also slightly soluble in alcohol. According Bhattacharya *et al.*, the salt is still present in an amorphous structure when it is calcined at 800 °C. It turns to η-Al_2_O_3_ at 900 °C and transforms to α-Al_2_O_3_ at 1100–1200 °C [12]. For hydrochloric acid with aluminum, hexahydrate aluminum trichloride AlCl_3_·6H_2_O is formed and decomposes to alumina at 300 °C [13].

Alumina exists in several metastable crystalline structures: η-, γ-, δ-, θ-, β-, κ-, χ, and α-alumina. Hexagonal α-alumina with lattice parameters of a = 4.758 Å and c = 12.991 Å is the most thermodynamically stable phase of alumina [14]. Calcinations of gibbsite Al(OH)_3_ show a series of decompositions and phase transformations of the aluminum compound towards the more stable phase at higher temperatures. As the calcination temperature increases, the series of transformations is Al(OH)_3_ → γ-AlOOH → γ-Al_2_O_3_ → δ-Al_2_O_3_ → θ-Al_2_O_3_ → α-Al_2_O_3_[15]. Factors such as particle size, heating rate, impurities, and atmosphere may influence the sequence of phase transformations of alumina due to the effect on the kinetics of transformation [14,16–18]. According to Gitzen [19], γ-Al_2_O_3_ transforms to δ-Al_2_O_3_ when calcined above 800 °C. The δ-Al_2_O_3_ transforms to θ-Al_2_O_3_ when calcined above 1000 °C. Finally, θ-Al_2_O_3_ transforms to α-Al_2_O_3_ when calcined above 1100 °C. However, the presence of impurities alters the barrier of phase transformations. For example, calcinations of γ-Al_2_O_3_ with 3% platinum impurity can cause the α-Al_2_O_3_ to form below 1100 °C [8,19,20].

The conversion of aluminum cans to alumina has not been reported in the ceramics processing literature. In this study, α-alumina was produced from waste aluminum cans. Beverage aluminum cans were chosen for this conversion process because they contribute a huge amount to solid waste in most countries. They consist primarily of aluminum with a small amount of other additional metals such as magnesium, manganese, iron, silicon and copper.

## 2. Results and Discussion

In the process of aluminum sulfate formation, the conditions within the digester (concentration, temperature and pressure) were set to optimize the reaction. This process revealed that the heated aluminum cans were converted into aluminum sulfate Al_2_(SO_4_)_3_ after being mixed with 8.0 M of sulfuric acid, H_2_SO_4_. The chemical reaction of aluminum with H_2_SO_4_ to form hydrogen gas and aluminum sulfate is shown in Equation 1.

(1) $$2\text{Al }(\text{s})+3{\text{H}}_{2}{\text{SO}}_{4}\;(\text{aq})\to 3{\text{H}}_{2}\;(\text{g})+{\text{Al}}_{2}{\left({\text{SO}}_{4}\right)}_{3}\;(\text{aq})$$

In the precipitation process, the white semi-liquid, aluminum sulfate Al_2_(SO_4_)_3_, was mixed with excess ethanol. The white precipitate formed was octadecahydrate aluminum sulfate, Al_2_(SO_4_)_3_·18H_2_O, salt, which is insoluble in ethanol. The white precipitate was filtered and rinsed with ethanol gently to remove excess acid on the salt. It was then dried in an oven at 120 °C for 16 h and crushed into smaller powder size. The XRD results for the white precipitate are shown in Figure 3. These results match the XRD references for Al_2_(SO_4_)_3_·18H_2_O, Al_2_(SO_4_)_3_·17H_2_O, and Al_2_(SO_4_)_3_·16H_2_O. The chemical reaction for Al_2_(SO_4_)_3_·18H_2_O formation from Al_2_(SO_4_)_3_ is shown in Equation 2.

(2) $${\text{Al}}_{2}{\left({\text{SO}}_{4}\right)}_{3}\;(\text{aq})+18{\text{H}}_{2}\text{O }(1)\to {\text{Al}}_{2}{\left({\text{SO}}_{4}\right)}_{3}\cdot 18{\text{H}}_{2}\text{O }(\text{s})$$

Alumina powders were produced from the conversion of the Al_2_(SO_4_)_3_·18H_2_O at high temperatures. Al_2_(SO_4_)_3_·18H_2_O is a complex molecule. Calcinations on the Al_2_(SO_4_)_3_·18H_2_O break down the bond in the complex molecule and provide sufficient interaction for the elements to form a new compound that is simpler and more stable. At sufficiently high calcination temperatures, Al_2_(SO_4_)_3_·18H_2_O is fully converted into alumina by eliminating water vapor, followed by the elimination of sulfur trioxide gas; these reactions are shown in Equation 3 and 4 [11,12,21]. Some volatile impurities in the gaseous state are also driven off. The calcinations provide heat energy to the alumina, which is needed for it to undergo further phase transformations to form α-alumina, the most stable crystalline structure of alumina.

(3) $${\text{Al}}_{2}{\left({\text{SO}}_{4}\right)}_{3}\cdot 18{\text{H}}_{2}\text{O }(\text{s})\to {\text{Al}}_{2}{\left({\text{SO}}_{4}\right)}_{3}\;(\text{s})+18{\text{H}}_{2}\text{O }(\text{g})$$

(4) $${\text{Al}}_{2}{\left({\text{SO}}_{4}\right)}_{3}(\text{s})\to {\text{Al}}_{2}{\text{O}}_{3}\;(\text{s})+3{\text{SO}}_{3}\;(\text{g})$$

**Figure 1:**
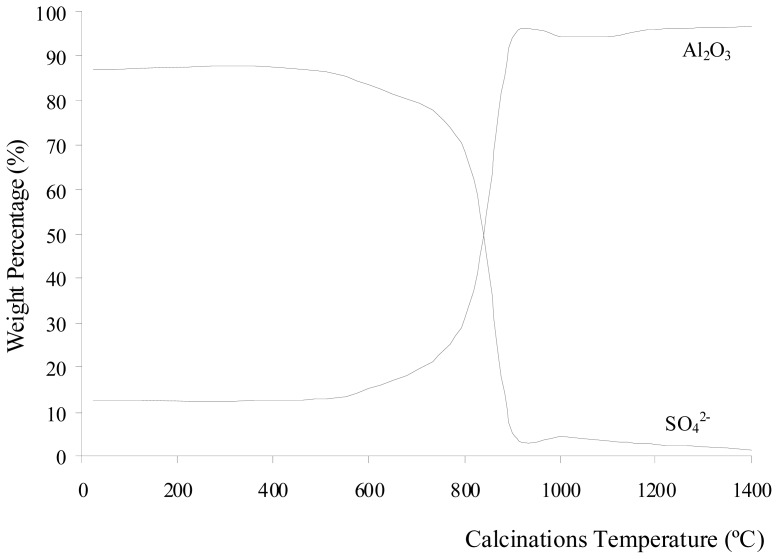
Weight percentage of aluminium oxide and sulfate ions present in Al_2_(SO_4_)_3_·18H_2_O before and after calcinations at 400–1400 °C for 3 h.

**Figure 2:**
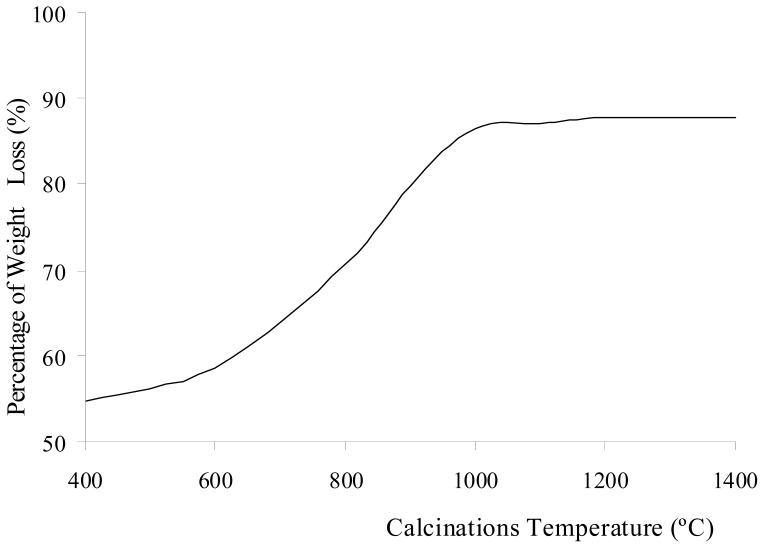
Percentage of weight loss of Al_2_(SO_4_)_3_·18H_2_O after calcinations at 400–1400 °C for 3 h.

For the commercialized product of aluminum cans, most of aluminum cans are made up of more than 97 percent aluminum. The remaining trace elements include one percent magnesium, one percent manganese and about one percent of iron, silicon and copper. XRF tests were performed on the Al_2_(SO_4_)_3_·18H_2_O before and after calcinations at 400–1400 °C for 3 h to determine the elements that present in the sample at each stage. The weight compositions of the elements which present in the sample before and after calcinations at 400–1400 °C for 3 h are tabulated in Table 1. Figure 1 shows the change in weight percentage due to aluminum oxide and sulfate ions present in Al_2_(SO_4_)_3_·18H_2_O before and after calcination at different temperatures. The percentage of weight loss during the calcinations of Al_2_(SO_4_)_3_·18H_2_O is shown in Figure 2. The weight loss increased with increasing calcination temperature. The loss of weight resulted from the water, sulfur trioxide and impurities in the Al_2_(SO_4_)_3_·18H_2_O salt being driven off during the calcinations. Each compound was eliminated at a different temperature stage. The diffusivity of water, sulfur trioxide and the impurities in the salt was also dependent on the calcination temperature. The diffusivity rate increased as the calcination temperature increased. The weight contribution of Al_2_O_3_ in Al_2_(SO_4_)_3_·18H_2_O before the calcinations was 12.51%, whereas SO_3_ contributed of 86.88%. When Al_2_(SO_4_)_3_·18H_2_O was calcined at 400 °C, the weight compositions were almost unchanged. However, there was a weight loss of 54.78% due to the elimination of ethanol and water below 400 °C. These compounds are made up of light elements that cannot be detected by XRF. The dehydration process of Al_2_(SO_4_)_3_·18H_2_O salt involved several phase transformations from high hydration phases to low hydration phases before being fully dehydrated into Al_2_(SO_4_)_3_. The hydrated salts that may have been present are Al_2_(SO_4_)_3_·16H_2_O, Al_2_(SO_4_)_3_·14H_2_O, Al_2_(SO_4_)_3_·9H_2_O, and Al_2_(SO_4_)_3_·5H_2_O [22,23]. Because there was no significant change in the presence of sulfate ions below 600 °C, the greater weight loss of Al_2_(SO_4_)_3_·18H_2_O below 600 °C was attributed to the dehydration of the salt.

Figure 1 shows that the sulfate ions were starts to decompose after being calcined at 600 °C. The weight due to the Al_2_O_3_ contribution increased as temperature increased. The sulfate ions being decomposed to sulfur trioxide and eliminated as a gas contributed to the increase in weight loss, as shown in Figure 2. The increase of calcination temperature causes the increase in weight loss. A pungent and colorless sulfur trioxide gas started to be released at this temperature. Figure 1 and 2 show that at calcinations of 800 °C, 900 °C, and 1000 °C, the samples had a large decrease in the weight contribution from sulfate ions and a large increase in the weight loss after each calcination process. These changes were due to a large amount of sulfur trioxide gas being eliminated as sulfate ions decomposed within these temperatures. At calcinations of 1000 °C, the percentage of weight loss achieved was 86.41%, and this percentage was almost constant when the sample was being calcined at higher temperatures. The weight contributions from aluminum oxide and sulfate ions were 94.30 and 4.58%, respectively. These numbers show that the sulfate ions were almost completely eliminated from the sample at 1000 °C. The residue of calcinations was alumina. The weight composition in Figure 1 shows that at calcinations above 1000 °C, there was a small decrease insulfate ion because a small amount of sulfur trioxide molecules diffused out from the alumina. In samples being calcined at 1400 °C, 1.18% of sulfate ions were detected. Hence, longer period for calcinations or higher calcination temperatures were required for all sulfur trioxide molecules to be diffused out from the alumina.

**Figure 3:**
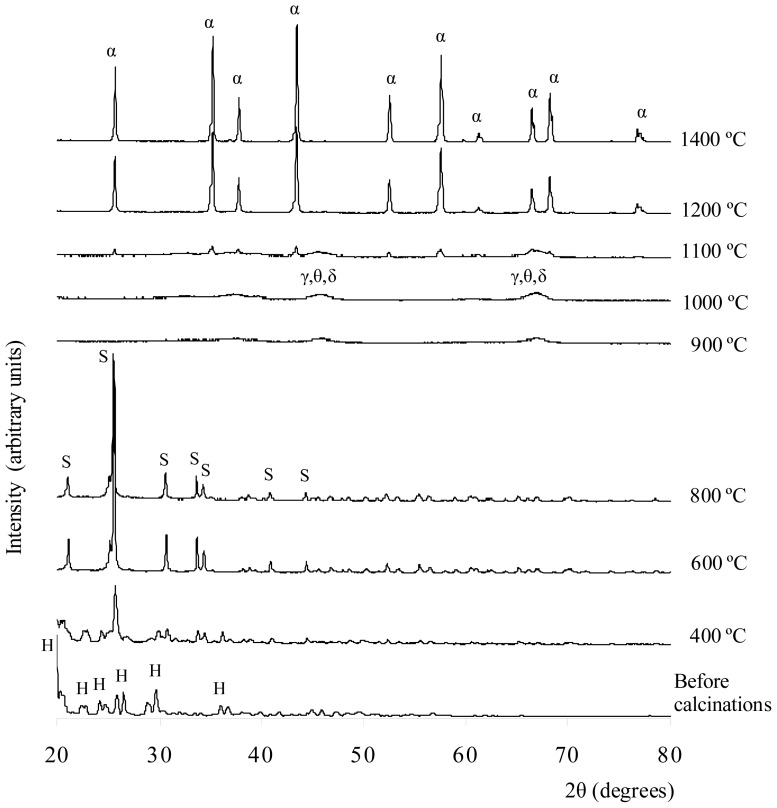
XRD result on Al_2_(SO_4_)_3_·18H_2_O sample before and after calcinations at 400–1400 °C for 3 h, where H = Al_2_(SO_4_)_3_·18H_2_O; S = aluminium sulfate; γ = γ-alumina; θ = θ-alumina; δ = δ-alumina; α = α-alumina.

Figure 3 shows the XRD results of the white solid formed by the precipitation method before and after being calcined at 400–1400 °C. The XRD result shows that before the calcinations, the sample matched the XRD reference of Al_2_(SO_4_)_3_·18H_2_O. For the sample calcined at 400 °C, the XRD results revealed the phase present was aluminum sulfate Al_2_(SO_4_)_3_. Some unknown peaks are present in the XRD plots. Hence, other crystalline phases, such as various types of hydrated aluminum sulfate or impurities, might have been present in the sample. Alumina was not produced at this temperature because it is relatively very low; therefore, it is unlikely that the sulfate ions decomposed. After being calcined at 600 °C, more aluminum sulfate phase peaks were matched with the ICSD code of 073249. The 2θ peaks (or Miller indices), which indicated that aluminum sulfate was present, were 21.0687° (104), 25.5532° (11-3), 30.7637° (024), 33.7308° (11-6), 40.9962° (303), 44.3888° (11-9), and 46.8175° (306). These peaks were also matched with the results reported by Khouzani *et al.*[24]. As the temperature increased from 400 °C to 600 °C, the intensity of aluminum sulfate phase peaks increased. The XRD results do not show that the sample was a hydrated salt with other impurities present as well. Therefore, water molecules and impurities might already have been removed at this temperature. At 800 °C, the XRD result for the sample indicates that the aluminum sulfate phase was present but at a relatively lower amount than at 600 °C. The structure of the aluminum sulfate at 600 °C and 800 °C was highly crystalline, and the crystal system was rhombohedral. After being calcined at 900°C, the sample lost its crystallinity, and small amorphous humps appeared at 2θ = 44°–47° and 65°–68°. These two humps were due to the interference of XRD signals of γ-, θ- and δ-alumina. Wood and Wilson [25], as well as Yang *et al.*[26], showed that γ-, θ- and δ-alumina peaks also existed in the range of 44°–47° and 65°–68°. At this temperature, the aluminum sulfate phases lose their bonding and appear highly amorphous in nature. The same phenomena can also be observed in the samples calcined at 1000 °C, but the intensity of the amorphous hump was increased. For the sample calcined at 1100 °C, a few small and sharp peaks of α-alumina started to emerge along with the amorphous hump compared to the XRD results for the 900 °C and 1000 °C calcinations. These results indicate that at this temperature, the alumina particles in several crystallized alumina were rearranged to form the α-alumina phase. The α-, γ-, δ-, and θ-alumina co-existed in discrete and short range order. The crystal structure of the α-alumina was crystalline, and the crystal system was rhombohedral. The sample calcined at 1200 °C was converted to α-alumina with a highly crystalline structure. This finding was confirmed by referring to the XRD reference database (ICSD collection code 025778). The crystal system of the structure was rhombohedral and had a density of 4.02 g cm^−3^ and lattice parameters of a = b = 4.7510 Å and c = 12.9700 Å. The XRD peaks that indicated the presence of α-alumina were at 2θ (Miller indices) = 25.594° (012), 35.197° (104), 37.804° (110), 43.381° (113), 52.588° (024), 57.538° (116), 59.782° (211), 61.333° (018), 66.547° (214), 68.230° and 76.909° (119). For the calcinations at 1400 °C, the sample showed the same pattern as the sample calcined at 1200 °C. The intensity of XRD peaks increased, indicating that more α-alumina crystals were distributed throughout the sample.

## 3. Experimental Section

The starting materials used were discarded aluminum cans, sulfuric acid (H_2_SO_4_), ethanol (CH_2_CH_3_OH) and distilled water. The aluminum cans were heated for 3 h at 600 °C in a Carbolite Electrical Furnace to remove the coating and impurities on their surface. The heated aluminum cans were then cut into small pieces to increase the surface area available to react with sulfuric acid solution. The heated aluminum cans were then weighed according to the stoichiometric proportions calculated using a Digital Analytical Balance with an accuracy of ±0.0001 g. Gonczy and Mitsche [9] used 3 M to 12 M of sulfuric acid in their investigation to prepare high-purity α-alumina from aluminum. In this study, 8.0 M of excess sulfuric acid solution, H_2_SO_4_, was chosen because the reaction was found to proceed at a steady state and a high rate. The H_2_SO_4_ solution was poured slowly over the heated aluminum pieces because the reaction occurred violently and a large amount of heat and gas with an unpleasant odor were released. A glass rod was used to stir the mixture moderately. The products were filtered using a Buchner funnel to obtain a white solution, which was aluminum sulfate Al_2_(SO_4_)_3_. Then, the acidic aluminum sulfate solution was precipitated by ethanol in a ratio of 2:3. The mixture was cooled to obtain a white precipitate. The white precipitate was rinsed with ethanol and then placed into an oven at 120 °C for 16 h for drying. The homogeneous powder was then calcined in an electric furnace for 3 h at 400–1400 °C; the heating and cooling rates were 10 °C min^−1^. The masses of the samples before and after the calcinations were recorded to determine the weight loss percentage.

The alumina samples were also tested with an XRF analyzer to determine the weight contribution of aluminum, sulfur and other elements in the samples. XRF is a non-destructive analytical technique used to identify and determine the content of element in the sample. In this study, the X-ray Fluorescence spectrometer EDX-720/800HS/900HS machine was used and the XRF results are presented as the weight percentage of the element and a possible oxide form for the element. The changes in the weight percentages of Al_2_O_3_ and SO_3_ were investigated to determine the effect of the calcinations on the hydrated aluminum sulfate.

The alumina samples were then characterized by an X-ray diffractometer (Rigaku, AX-2500) using 0.1542 nm Cu K_α_ to identify the phases present at different calcination temperatures. Data reduction routines rapidly determined the peak position, relative intensities, and calculate intra-crystalline d-spacing. The identification of unknown crystalline materials was completed using the complete ICSD powder diffraction files.

## 4. Conclusions

Alumina, Al_2_O_3,_ was produced from waste aluminum through a reaction with concentrated sulfuric acid, precipitation of the resulting solution and calcinations of the precipitate. The sample produced from the precipitation was Al_2_(SO_4_)_3_·18H_2_O. The samples before and after calcination were characterized using XRF and XRD. The XRF tests show that the weight percentage of alumina increased and sulfate ions decreased as the calcination temperature increased. The maximum weight percentage of alumina achieved by the calcinations was 96.79% at 1400 °C. This production method of α-Al_2_O_3_ from aluminum waste is practical for industry because a large quantity of highly crystalline α-Al_2_O_3_ can be produced. In addition, the production cost is low because the process requires only discarded aluminum cans, sulfuric acid and ethanol.

Calcinations of hydrated aluminum sulfate Al_2_(SO_4_)_3_·18H_2_O at high temperatures eliminate steam, sulfur trioxide and volatile impurities. Calcinations dehydrate the Al_2_(SO_4_)_3_·18H_2_O salt and breakdown the sulfate ions at high temperatures to produce α-alumina. Water molecules are released from the salt below 600 °C, whereas the SO_3_ gas is released from the salt within 600 °C to 1000 °C. The alumina is transformed to α-alumina after calcinations at 1200 °C. The results of the XRD analysis show that the α-alumina (α-Al_2_O_3_) phase existed after the sample had been calcined at 1200 °C or and above. The increase of calcination temperature causes the increase in weight loss. The percentage of weight loss increased as the calcination temperature increased, but the weight loss was almost constant for 1000 °C and above.

## Figures and Tables

**Table 1:** Weight percentage of oxides present in Al_2_(SO_4_)_3_·18H_2_O before and after calcinations at 400–1400 °C for 3 h. BC = before calcination.

Calcination Temperature (°C)	Al_2_O_3_	SO_4_^2−^	SiO_2_	ZnO	MnO	Fe_2_O_3_	CuO
BC	12.51	86.88	0.30	0.12	0.10	0.07	0.02
400	12.31	87.33	0.09	0.01	0.14	0.12	-
600	16.31	83.38	0.08	0.02	0.12	0.09	-
800	31.19	68.28	0.02	0.02	0.24	0.22	0.03
900	94.77	3.52	0.02	0.10	0.70	0.84	0.05
1000	94.30	4.58	0.01	-	0.73	0.38	-
1100	95.44	2.06	1.03	-	0.72	0.75	-
1200	96.03	1.62	1.08	0.14	0.65	0.48	-
1400	96.79	1.18	0.71	0.11	0.64	0.57	-
